# Dephasing Process of a Single Atom Interacting with a Two-Mode Field

**DOI:** 10.3390/e23020252

**Published:** 2021-02-22

**Authors:** Eied M. Khalil, Kamal Berrada, Sayed Abdel-Khalek, Beida Alsubei, Hichem Eleuch

**Affiliations:** 1 Department of Mathematics and Statistics, College of Science, Taif University, P.O. Box 11099, Taif 21944, Saudi Arabia; eiedkhalil@tu.edu.sa (E.M.K.); sabotalb@tu.edu.sa (S.A.-K.); B.bmta@tu.edu.sa (B.A.); 2 Physics Department, College of Science, Imam Mohammad Ibn Saud Islamic University (IMSIU), Riyadh 13318, Saudi Arabia; 3 Department of Applied Physics and Astronomy, University of Sharjah, Sharjah27272, United Arab Emirates; hichemeleuch@yahoo.fr; 4 Department of Applied Sciences and Mathematics, College of Arts and Sciences, Abu Dhabi University, Abu Dhabi 59911, United Arab Emirates; 5 Institute for Quantum Science and Engineering, Texas A&M University, College Station, Texas, TX 77843, USA

**Keywords:** qubit-field system, finite dimensional pair coherent states, phase damping, linear entropy, Fisher information entropy squeezing

## Abstract

We consider the interaction of a qubit system with a two-mode field in the presence of multi-photon transition and phase damping effect. We use the master equation to obtain the density operator when the qubit is initially prepared in its excited state and the field is in a finite-dimensional pair coherent state. The properties of the considered system, such as the population inversion, amount of the mixedness, parameter estimation, and squeezing, are explored for one- and two-photon transitions. The effects of photon addition to the field and phase damping on the evaluation of these quantumness measures are also investigated.

## 1. Introduction

In quantum optics theory, the model of Jaynes and Cummings (JC) is a fully solvable model illustrating the interaction between a qubit system and a cavity field in the presence of a rotating-wave approximation [1]. The JC model was verified experimentally [2] and has numerous extensions, such as two atoms [3] and a bimodal cavity field [4]. In addition, many non-intuitive predictions, such as the phenomena of collapse and revival [5], anti-bunching [6], chaos [7], squeezing [8], and trapping state were studied [9]. The usual JC model was applied to examine the interaction of two atoms and multi-mode fields [10,11,12,13,14,15,16]. The importance of this system has increased due to advances in quantum information theory. The atomic inversions, phase distribution, quasi-probability function, phase distribution, and quantum entanglement for the case of the multi-photon JC model were discussed and analyzed [17]. In recent years, the generation and characterization of the entanglement between atoms inside a resonant microcavity have been discussed [18]. The entanglement features of atomic systems in stochastic interaction with a quantized field in a cavity QED were investigated using the JC model [19].

In order to measure how pure a given density operator is, quantifiers of purity exist, such as the von Neumann entropy or the linear entropy [20,21]. Entropy is a widely used concept in various fields. In fact, the degree of mixedness that would reflect, among other things, how the atom and the field were entangled or how strong the interaction was with the environment, is an important challenge and goal that need to be well defined. Entropy is perhaps the most popular measure to estimate the mixedness degree of a given state [22,23,24,25]. It characterizes precisely the purity of a given state depending on the Hilbert space dimension. The degree of mixedness needs to be determined in each problem. The reason for this is that for certain density operators, it is necessary to have a priori information.

In recent years, atomic squeezing has generated much interest due to its potential applications in high-resolution atomic fountain clocks [26], high-resolution spectroscopy [27], generation of squeezed light [28], high-precision rotational polarization measurements [29], and so on. The concept of the squeezed states for atoms was firstly established by Wodkiewicz and others [30]. Walls and Zoller demonstrated that squeezed fluorescence can be emitted by a squeezed atom in a coherently driven qubit system [28]. Agarwal and Puri investigated the atomic squeezing in a quantum system of multi-qubits damped by a broadband squeezed vacuum. Spin and projection noise in spectroscopy was investigated by Wineland et al. [26] and several works focused on the atomic squeezing predicted by the JC model [31]. Recently, attention has been drawn to the compression in a set of atoms illuminated by squeezed light, including atomic spin polarization and quantum noise [32], and the few quantum states of photons generated from the squeezed atoms [33]. All these aforementioned studies on atomic squeezing explored the Heisenberg uncertainty relation (HUR), which is considered as a limitation on the measurements of the fluctuations. The HUR was formulated as a function of the standard deviations (or variances) of the observables. The considered aspects are generally the most appropriate measures of the underlying uncertainty associated with the fluctuations.

Quantum coherence is known to play a role in a more accurate estimation of an unknown parameter provided by classical deterministic physics. Quantum metrology permits us to achieve a measurement accuracy that exceeds the limit we can achieve with classical measurements by utilizing quantum features and it will become one of the pillars of quantum sensors in the future [34]. Using N probes in parallel, the Heisenberg limit can be achieved in the absence of noise [35,36,37,38,39]. The Fisher information, which determines the sensitivity according to the changes in the parameter estimation, is at the heart of quantum parameter estimation theory. This quantity uses a bound to distinguish the set of members from probability distributions. A higher value of Fisher information means that the precision in estimating a parameter is higher. However, decoherence resulting from noise can limit the accuracy of the result of the parameter estimation and results in a loss of entanglement or coherence of the probes [40,41,42]. It is therefore of interest to preserve the Fisher information from the decoherence. From this point of view, many studies have been devoted to the search for strategies to protect and control the Fisher information in the presence of external noises [43,44,45,46,47].

A realistic quantum system necessarily interacts with the environment around it. This spontaneous interaction essentially results in the destruction of the coherence stored in the quantum system, i.e., the so-called decoherence [48]. Typically, the interactions caused by the environment induce entangled states of the system-environment ensemble. Therefore, the entanglement that builds up during the interaction can be considered as a primary mechanism on which the decoherence is based. In this way, the control and manipulation of the decoherence can be exploited to enhance the entanglement between the system and its environment. It is proven that the entanglement of the induced steady state between a two-level atom and its spontaneous emission excitation is manipulated by detuning the intensity and relative phase of the fields [49,50,51,52,53,54]. It is worth noting that the hybrid entanglement between a photon and an atom has applications in quantum tools, such as quantum networks [55], quantum repeater [56,57], and so on.

In this paper, we aim to extend previous studies on field-atom systems to investigate the case of a qubit system with a two-mode field in the presence of multi-photon transition and phase damping (PD) effect. We introduce the master equation to obtain the density matrix of the system when the qubit is initially prepared in its excited state and the field is in a finite-dimensional pair coherent state (FDPCS). We examine the properties of the considered system, such as the population inversion, amount of the mixedness, parameter estimation, and squeezing for one- and two-photon transitions. We explore the effects of photon addition to the field and phase damping on the evaluation of these quantumness measures.

The present manuscript is structured as follows. In Section 2 we introduce the model and present an explicit expression of the evolved density operator. Section 3 is dedicated to a discussion on the dynamics of the population inversion, qubit purity, parameter estimation, and squeezing. Finally, Section 4 summarizes the main results.

## 2. Model and Solution

The proposed quantum system consists of a qubit that interacts with a two-mode field in the presence of multi-photon transition and the phase damping effect.

Let us first discuss the effects of the damping effect on a system which contains an N-level atom represented by SU(2) Lie algebra operators interacting with a qubit. The system Hamiltonian can be written as [58,59,60,61]

(1) $$\frac{\hat{H}}{\hbar }=\frac{\Omega }{2}{\hat{\sigma }}_{z}+\omega {\hat{J}}_{z}+\varepsilon ({\hat{\sigma }}_{+}{\hat{J}}_{-}^{k}+{\hat{\sigma }}_{-}{\hat{J}}_{+}^{k}),$$

where ${\hat{J}}_{-}$ and ${\hat{J}}_{+}$, respectively, denote the lowering and raising operators of the SU(2) system that satisfy the following commutation relation:

$$[{\hat{J}}_{-},\;{\hat{J}}_{+}]=-2{\hat{J}}_{z},\;[{\hat{J}}_{z},\;{\hat{J}}_{\pm }]=\pm {\hat{J}}_{\pm }.$$

The parameters ω and Ω are, respectively, the N-level atomic frequency and atomic frequencies, while ε is the coupling parameter and k is an integer number. ${\hat{\sigma }}_{+}({\hat{\sigma }}_{-})$ and ${\hat{\sigma }}_{z}$ indicate the transition operators of the qubit that satisfy the commutation relations

(2) $$[{\hat{\sigma }}_{z},\;{\hat{\sigma }}_{\pm }]=\pm 2{\hat{\sigma }}_{\pm },[{\hat{\sigma }}_{+},\;{\hat{\sigma }}_{-}]={\hat{\sigma }}_{z}.$$

We use the Schwinger angular-momentum operators [62]. Considering two independent bosonic modes described by annihilation/creation operators $\hat{A}$ and $\hat{B}$, the Schwinger angular-momentum operators take the following relation:

(3) $$J_{x}=\frac{\left({\hat{A}}^{†}\hat{B}+{\hat{B}}^{†}\hat{A}\right)}{2},\;J_{y}=\frac{\left({\hat{A}}^{†}\hat{B}-{\hat{B}}^{†}\hat{A}\right)}{2i},\;J_{z}=\frac{\left({\hat{A}}^{†}\hat{A}-{\hat{B}}^{†}\hat{B}\right)}{2},$$

which formulates the generators of the SU(2) Lie algebra and satisfies

(4) $$[J_{x},\;J_{y}]=iJ_{z},\;[J_{y},\;J_{z}]=iJ_{x}\;\mathrm{and}\;[J_{z},\;J_{x}]=iJ_{y}.$$

Considering the realization

(5) $$J_{+}=J_{x}+iJ_{y}={\hat{A}}^{†}\hat{B},\;J_{-}=J_{x}-iJ_{y}={\hat{B}}^{†}\hat{A},$$

which is just the square of the total angular momentum and commutes with all the generators of the Lie algebra. Therefore, the Hamiltonian (1) becomes

(6) $$\frac{\hat{H}}{\hbar }=\frac{\Omega }{2}{\hat{\sigma }}_{z}+\frac{\omega }{2}({\hat{A}}^{†}\hat{A}-{\hat{B}}^{†}\hat{B})+\varepsilon ({\hat{\sigma }}_{+}{\hat{B}}^{†k}{\hat{A}}^{k}+{\hat{\sigma }}_{-}{\hat{A}}^{†k}{\hat{B}}^{k}).$$

In order to interpret the physical phenomena associated with the Hamiltonian (6), we introduce the differential equations by applying the Heisenberg equations of motion

(7) $$\begin{array}{c} \frac{d{\hat{A}}^{†}\hat{A}}{dt}=i\varepsilon k({\hat{\sigma }}_{+}{\hat{B}}^{†k}{\hat{A}}^{k}-{\hat{\sigma }}_{-}{\hat{A}}^{†k}{\hat{B}}^{k})\\ \frac{d{\hat{B}}^{†}\hat{B}}{dt}=-i\varepsilon k({\hat{\sigma }}_{+}{\hat{B}}^{†k}{\hat{A}}^{k}-{\hat{\sigma }}_{-}{\hat{A}}^{lk}{\hat{B}}^{k})\\ \frac{d{\hat{\sigma }}_{z}}{dt}=-2i\varepsilon ({\hat{\sigma }}_{+}{\hat{B}}^{†k}{\hat{A}}^{k}-{\hat{\sigma }}_{-}{\hat{A}}^{†k}{\hat{B}}^{k}), \end{array}$$

from which we can show that

(8) $$\begin{array}{l} \hat{C}={\hat{A}}^{†}\hat{A}-{\hat{B}}^{†}\hat{B}+k{\hat{\sigma }}_{z},\\ \hat{Q}={\hat{A}}^{†}\hat{A}+{\hat{B}}^{†}\hat{B}, \end{array}$$

where $\hat{C}$ and $\hat{Q}$ are constants of motion. Then, the Hamiltonian system (1) becomes

(9) $$\frac{\hat{H}}{\hbar }=\omega \hat{C}+\hat{D},$$

where the operator $\hat{D}$ takes the form

(10) $$\hat{D}=\frac{\delta {\hat{\sigma }}_{z}}{2}+\lambda ({\hat{\sigma }}_{+}{\hat{B}}^{†k}{\hat{A}}^{k}+{\hat{\sigma }}_{-}{\hat{A}}^{†k}{\hat{B}}^{k}),$$

and the quantity δ denotes the detuning function which can be expressed as

(11) δ=(Ω−kω).

We consider that the qubit starts from its excited state |e〉, while the field is prepared in the FDPCS given by [63]

(12) $$|\zeta ,\;q\rangle =N_{q}\overset{q}{\underset{n=0}{\sum }}\zeta^{n}\sqrt{\frac{(q-n)!}{q!n!}}|q-n,\;n\rangle$$

The normalization factor $N_{q}$ has the form

(13) $$N_{q}={\left[\overset{q}{\underset{n=0}{\sum }}{\left|\zeta \right|}^{2n}\frac{(q-n)!}{q!n!}\right]}^{-\frac{1}{2}}={\left({\;}_{1}F_{0}(-q,-|\zeta {|}^{2})\right)}^{-\frac{1}{2}},$$

where ${}_{1}F_{0}$ is a generalized hypergeometric function. Note that |ζ, q〉 is an eigenstate of the pair operator $\left({\hat{A}}^{†}B+\frac{\zeta^{q+1}{\left(\hat{A}{\hat{B}}^{†}\right)}^{q}\;}{{\left(q!\right)}^{2}}\right)$ and the sum of the operator numbers $\left({\hat{A}}^{†}\hat{A}+{\hat{B}}^{†}\hat{B}\right)$:

$$\left({\hat{A}}^{†}B+\frac{\zeta^{q+1}{\left(\hat{A}{\hat{B}}^{†}\right)}^{q}}{{\left(q!\right)}^{2}}\right)\left|\zeta ,q\right.=\zeta \left|\zeta ,q\right.$$

$$\left({\hat{A}}^{†}\hat{A}+{\hat{B}}^{†}\hat{B}\right)\left|\zeta ,q\right.=q\left|\zeta ,q\right..$$

We introduce the phase damping terms to write the master equation for the system Hamiltonian at zero temperature through the transformation $\hat{W}(t)=\exp(i\hat{H}t)\hat{\rho }(t)\exp(i\hat{H}t)$ [64]:

(14) $$\frac{d\hat{\rho }(t)}{dt}=\gamma \left[2{\hat{J}}_{z}\hat{\rho }(t){\hat{J}}_{z}-\hat{\rho }(t){\hat{J}}_{z}^{2}-{\hat{J}}_{z}^{2}\hat{\rho }(t)\right].$$

After using the transformations (3), Equation (14) becomes

(15) $$\frac{d\hat{\rho }(t)}{dt}=\gamma \left[2\left(\frac{{\hat{A}}^{†}\hat{A}-{\hat{B}}^{†}\hat{B}}{2}\right)\;\hat{\rho }(t)\left(\frac{{\hat{A}}^{†}\hat{A}-{\hat{B}}^{†}\hat{B}}{2}\right)-\hat{\rho }(t){\left(\frac{{\hat{A}}^{†}\hat{A}-{\hat{B}}^{†}\hat{B}}{2}\right)}^{2}-{\left(\frac{{\hat{A}}^{†}\hat{A}-{\hat{B}}^{†}\hat{B}}{2}\right)}^{2}\hat{\rho }(t)\right].$$

After eliminating quantities with rapid oscillations, the differential Equation (15) takes the following form

(16) $$\begin{array}{c} \frac{dR(t)}{dt}=\frac{\gamma }{2}\sum_{m=0}^{q}\sum_{n=0}^{q}\left\{\begin{array}{c} \left(2(q-2n)+1\right)\;\left(2(q-2m)+1\right)[\Gamma_{nn}^{++}\hat{W}(t)\Gamma_{mm}^{++}+\Gamma_{nn}^{--}\hat{W}(t)\Gamma_{mm}^{--}\\ +\Gamma_{nn}^{++}\hat{W}(t)\Gamma_{mm}^{--}+\Gamma_{nn}^{--}\hat{W}(t)\Gamma_{mm}^{++}]\\ +\Gamma_{nn}^{+-}\hat{W}(t)\Gamma_{mm}^{-+}e^{2i\mu_{nm}}+\Gamma_{nn}^{-+}\hat{W}(t)\Gamma_{mm}^{+-}e^{-2i\mu_{nm}} \end{array}\right\}\;\\ -\frac{\gamma }{4}\sum_{n=0}^{q}\left[{\left(2(q-2n)+1\right)}^{2}+1\right]\;\left\{\left(\Gamma_{nn}^{++}+\Gamma_{nn}^{--}\right)\;\hat{W}(t)+\hat{W}(t)\left(\Gamma_{nn}^{++}+\Gamma_{nn}^{--}\right)\right\} \end{array}$$

where $\Gamma_{nn}^{++}=|\phi_{n}^{J}\rangle \langle \phi_{n}^{L}|,\;\forall \;J,\;L=+,-,$ $|\varphi_{n}^{\pm }\rangle$ are the eigenstates of the Hamiltonian (6), $\mu_{nm}=\mu_{n}-\mu_{m}.$ The general solution takes the following form:

(17) $$\left(\begin{array}{c} |\phi_{n}^{+}\rangle \\ |\phi_{n}^{-}\rangle \end{array}\right)=\left(\begin{array}{cc} \cos\Theta_{n} & -\sin\Theta_{n}\\ \sin\Theta_{n} & \cos\Theta_{n} \end{array}\right)\;\left(\begin{array}{c} |q-n,\;n,\;e\rangle \\ |q-n+1,\;n+1,\;g\rangle \end{array}\right),$$

with $\Theta_{n}=\tan^{-1}(\frac{\delta^{2}}{4}+\mu_{n}^{2})$ and $\mu_{n}=\lambda \sqrt{\frac{(q-n+k)!}{(q-n)!}\frac{n!}{(n-k)!}}.$

## 3. Numerical Results

By using the numerical solution for (16), we study the effects of the photon number as well as the dephasing on the quantumness measures. This explains the main features of the considered system including the phenomena of collapse and revival, the qubit purity, the parameter estimation precision, and the phenomenon of entropy squeezing.

### 3.1. Population Inversion

The phenomena of collapse and revival are among the main tools in revealing the characteristics of a quantum system because of their close connection with the entanglement between the quantized field and the qubit. Moreover, we discuss the effect of the number of photons and the dephasing parameter on these phenomena through the equation

(18) $$W(t)=\frac{\rho_{ee}(t)-\rho_{gg}(t)}{2}.$$

**Figure 1:**
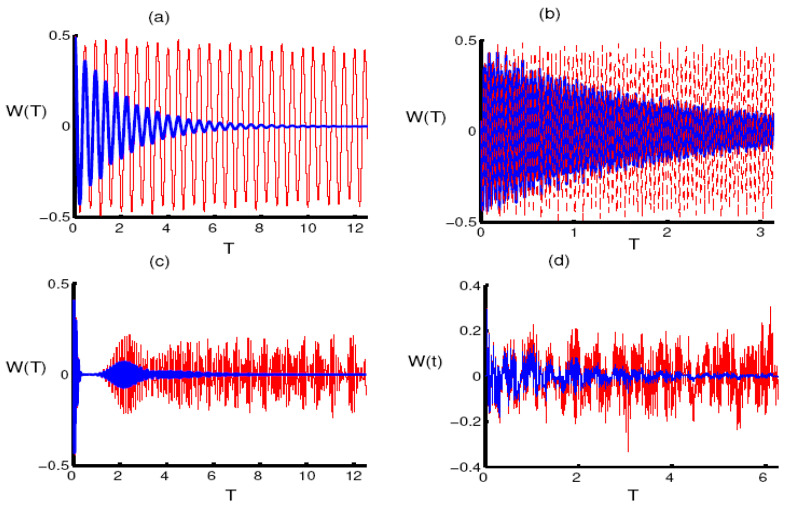
The evolution of the atomic inversion W(T) as a function of the dimensionless time T=εt. The radiation field (RF) is initially in a finite-dimensional pair coherent state (FDPCS) for ξ=30 and the single qubit is in its upper state. Plots (**a**,**c**) are for one-photon processes with k=1 and the two-photon processes with k=2 are shown in (**b**,**d**). The parameter q=50 is used in (**a**,**b**) and q=100 in (**c**,**d**). The red curve indicates the absence of the phase damping (PD) effect (γ/ε=0) and the blue curve indicates the presence of the PD effect (γ/ε=0.5).

Assume that the quantized field is described in the FDPCS (12) with a parameter q, while the qubit starts the interaction from the excited state. For the one-photon case, the dephasing parameter is neglected and the parameter q is assumed to be small. The function W(t) randomly oscillates between the excited state and the ground state and the collapse phenomenon does not materialize during the considered interaction period. After the dephasing of the interaction is included, the amplitude of the oscillations decreases and the function W(t) goes into a steady state after a short interval, as shown in Figure 1a. In the case of two photons, both the randomness and the intensity of the oscillations are significantly stronger. After adding the decay, the oscillations gradually collapse until they reach stability after a longer interval compared to the previous case, see Figure 1b. After increasing the parameter q and neglecting the decay terms, the preceding chaos becomes regular and fully shows the phenomena of collapse and revival. Moreover, the amplitude of the fluctuations decreases compared to the previous case, see Figure 1c. In the case of two photons, the oscillations return again to randomness, the phenomena of collapse and revival disappear, and the intensity of the oscillations decreases. After the dephasing parameter is considered, an internal energy is generated that restricts the movement of the qubit and causes it to rapidly collapse toward a steady state, as shown in Figure 1d.

### 3.2. Linear Entropy

In quantum mechanics theory, and especially quantum information theory, the linear entropy or impurity that measures the degree of mixedness is a scalar defined as

(19) $$L(t)=1-Tr\left(\rho_{q}^{2}(t)\right),$$

where $\rho_{q}(t)$ is the density matrix of the qubit system obtained by performing the trace over the two-mode field state. The linear entropy can range between zero, for a completely pure state, and 1/2, for a completely mixed state of the qubit. 

**Figure 2:**
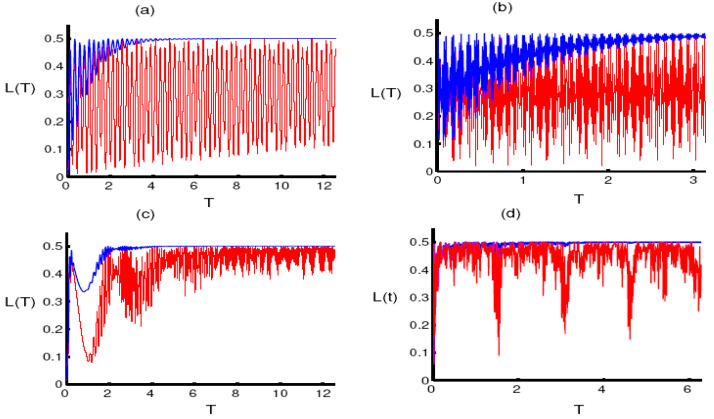
The evolution of the linear entropy L(T) as a function of the dimensionless time T=εt for the same conditions and parameters of Figure 1.

We begin the process of analyzing the evolution of the mixedness of the qubit state under the same conditions as before. In the case of small values of the parameter q, the linear entropy oscillates smoothly, reflecting the weakening of the amount of the mixedness of the qubit state. After taking into account the dephasing parameter, the oscillations of the linear entropy gradually become restricted upwards, and the previous amount tends to attain the maximum value 1/2 corresponding to a maximally mixed state of the qubit system. In the two-photon case, the intensity of the oscillations of the linear entropy increases with time corresponding to the case of a mixed state. More precisely, the function L(t) needs more time to reach the steady state L=1/2, in the presence of decoherence. In the presence of phase damping, the linear entropy of the qubit state reaches the maximum value before and after the mid-revival period in the population inversion. These results reflect the important role of the photon transition k, during the interaction and the photon number q, on the evolution of the mixedness. After the phase damping is considered, a potential force arises that transforms the qubit state from a completely pure state into a maximally mixed state (see Figure 2).

### 3.3. Quantum Fisher Information

The Fisher information of the qubit for a given process with a parameter ϕ is introduced as [35,36,37,38,39]

(20) $$F_{q}(t)=\mathrm{Tr}\left({\hat{\rho }}_{q}(\phi )L{\left(\phi \right)}^{2}\right),$$

where L(ϕ) is an operator satisfying

(21) $$\frac{\partial {\hat{\rho }}_{q}(\phi )}{\partial \phi }=\frac{1}{2}\left(L{\hat{\rho }}_{q}(\phi )+{\hat{\rho }}_{q}(\phi )L\right),$$

The spectral decomposition of the qubit state is given as

(22) $${\hat{\rho }}_{q}^{j}(\phi )=\underset{j}{\sum }\lambda_{j}(\phi )|j\rangle \langle j|.$$

Therefore, the Fisher information of the qubit density operator is given by

(23) $$F_{q}(t)=\underset{j}{\sum }\frac{{\left(\partial_{\phi }\lambda_{j}(\phi )\right)}^{2}}{\lambda_{j}(\phi )}+2\underset{j,\;j^{\prime }}{\sum }\frac{\left(\lambda_{j}(\phi )-\lambda_{j^{\prime }}(\phi )\right)}{\left(\lambda_{j}(\phi )+\lambda_{j^{\prime }}(\phi )\right)}|\langle j|\partial_{\phi }j^{\prime }\rangle {|}^{2}.$$

The first term of the last equation describes the classical Fisher information and the second one defines its quantum counterpart.

**Figure 3:**
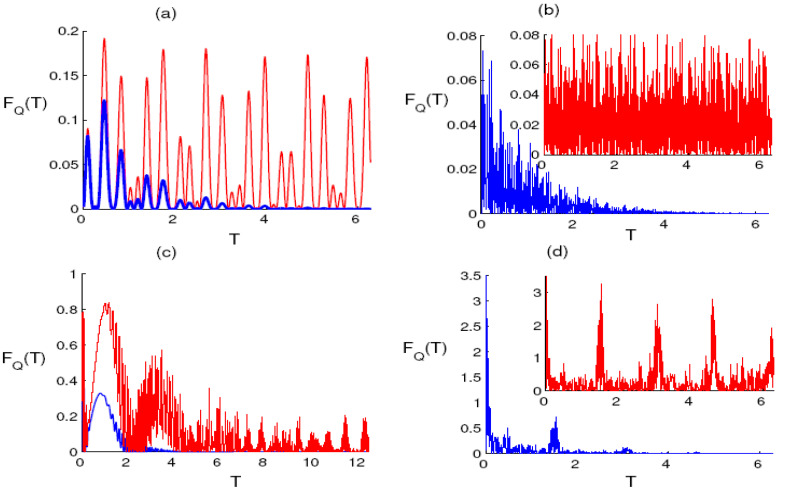
The evolution of the quantum Fisher information $F_{q}(T)$ as a function of the dimensionless time T=εt for the same conditions and parameters of Figure 1.

The Fisher information gives an indication of the amount of the mixedness of the qubit state. For the one-photon case, the decay parameter is neglected and the parameter q is assumed to be small. The function $F_{q}$ exhibits oscillations and ranges between 0 and 0.2. This indicates that the precision of the parameter estimation may be enhanced or restrained and that the qubit state is a mixed state during the interaction periods as seen in Figure 3a. In the case of two photons, the Fisher information decreases during the evolution. On the other hand, the intensity of the oscillations increases and the amplitude of the oscillations decreases significantly in presence of the phase damping effect, where the qubit state will be in a maximally mixed state at significantly large times. For large values of the parameter q, the situation is completely different, resulting in a strong correlation between the two qubits(see Figure 3b). The previous random oscillations become regular, the Fisher information reaches its maximum value and decrease significantly until it reaches the steady state. After the decoherence effect is considered, the value of the Fisher information quickly collapses and the function $F_{q}$ reaches a steady state with maximally mixed state of the qubit after a short time, as seen in Figure 3c. In the case of two photons, the precision of the parameter estimation is greater than that of the one photon case, and the function $F_{q}$ reaches the maximum value periodically. After the phase damping effect is taken into account, the oscillations of the Fisher information become quickly erased and dissipated after a short period of time (see Figure 3d).

### 3.4. Squeezing Phenomena

It is well known that all studies of atomic squeezing rely on the Heisenberg uncertainty inequality, which is the standard limit for measurements of quantitative fluctuations [65]. The formula is based on standard deviations of physical factors within the quantum system. These physical quantities are the most natural measures for the fundamental uncertainty dependent on quantum fluctuations [66,67]. Therefore, we study the squeezing entropy, which is known by the following formula,

(24) $$E({\hat{\sigma }}_{i})=\delta H({\hat{\sigma }}_{i})-\frac{2}{\sqrt{\delta H({\hat{\sigma }}_{z})}},$$

where $H({\hat{\sigma }}_{i})$ is the Shannon entropy for i=x or y.

**Figure 4:**
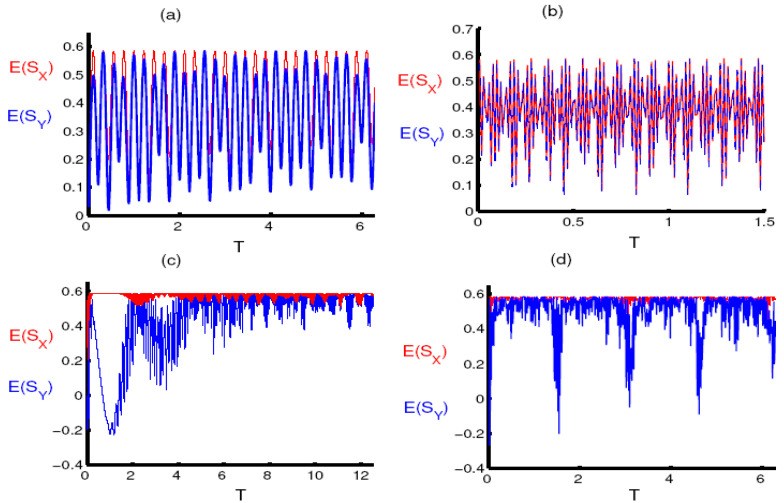
The evolution of the entropy squeezing components $E({\hat{S}}_{X})$ (red curve) and $E({\hat{S}}_{Y})$ (blue curve) as a function of the dimensionless time T=εt in absence of the PD effect γ/ε=0. The RF is initially in a FDPCS for ξ=30 and the single qubit is in its upper state. (**a**,**c**) for one-photon processes k=1 and for the two-photon processes k=2 in (**b**,**d**). The parameter q=50 is used in (**a**,**b**) and q=100 in (**c**,**d**).

Now, we consider the situation when the squeezing is achieved according to the conditions mentioned in the population inversion. Generally for small values of the parameter q there is no squeezing for either the component $E({\hat{\sigma }}_{x})$ or $E({\hat{\sigma }}_{y}).$ While the functions $E({\hat{\sigma }}_{x})$ and $E({\hat{\sigma }}_{y})$ oscillate and approach zero at many points, they do not become negative as shown in Figure 4a,b. For large values of the parameter q, squeezing is achieved in two periods, the first after the start of the interaction for a short period, and the second during the collapse period. The function $E({\hat{\sigma }}_{y})$ reaches the maximum value of squeezing at the midpoint of the collapse period, as shown in Figure 4c. The periods of squeezing decrease after taking into account the two-photon case, as observed in Figure 4d.

**Figure 5:**
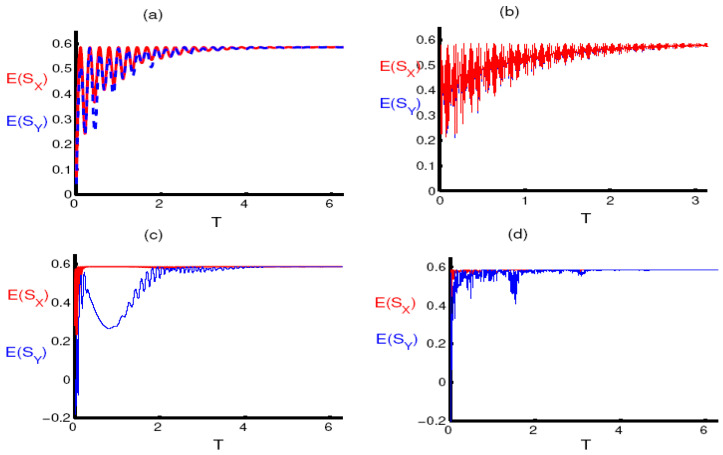
The same as Figure 4 under the effect of PD for γ/ε=0.5.

The phenomenon of squeezing in the $E({\hat{\sigma }}_{x})$ and $E({\hat{\sigma }}_{y})$ components completely disappears after considering the phase damping, and the amplitude of the oscillations gradually decreases until the function components are fixed after a period of time, as shown in Figure 5.

## 4. Conclusions

In summary, we considered the interaction of a qubit system with a two-mode field in the presence of multi-photon transition and phase damping effect. We used the master equation to obtain the density operator when the qubit system was initially prepared in its excited state and the field in a finite-dimensional pair coherent state. We examined the properties of this system, such as the population inversion, amount of the mixedness, parameter estimation, and squeezing for one- and two-photon transitions. We explored the effects of photon addition to the field and phase damping on the evaluation of these quantumness measures. We showed the collapse and revival phenomena of the population inversion according to the choice of the main physical parameters. We examined the time evolution of the mixedness of the qubit state through the linear entropy and showed situations in which the qubit state reaches the maximally entangled state. We examined the parameter estimation through the Fisher information that can be considered as an indication of the amount of the mixedness of the qubit state. Finally, the qubit squeezing was estimated through the entropy squeezing and the situation, in which the squeezing occurred with respect to the values of the physical parameters.

## Data Availability

Not applicable.
